# Protocol for a process evaluation of an external pilot cluster randomised controlled trial of a theory-based intervention to improve appropriate polypharmacy in older people in primary care: the PolyPrime study

**DOI:** 10.1186/s13063-021-05410-6

**Published:** 2021-07-14

**Authors:** Audrey Rankin, Gerard J. Molloy, Cathal A. Cadogan, Heather E. Barry, Ashleigh Gorman, Cristín Ryan, Alan Ferrett, Pat McCarthy, Gerard J. Gormley, Tom Fahey, Carmel M. Hughes, On behalf of the PolyPrime team

**Affiliations:** 1 School of Pharmacy, Queen’s University, 97 Lisburn Road, Belfast, BT9 7BL UK; 2 School of Psychology, National University of Ireland, Galway, Ireland; 3 School of Pharmacy and Pharmaceutical Sciences, Trinity College Dublin, Dublin, Ireland; 4 Public Involvement Enhancing Research, Belfast, Northern Ireland; 5 Donegal Volunteer Centre, Letterkenny, Ireland; 6 School of Medicine, Dentistry and Biomedical Sciences, Queen’s University Belfast, Belfast, UK; 7 Department of General Practice, Royal College of Surgeons in Ireland, Dublin, Ireland

**Keywords:** Polypharmacy, Behaviour change, Primary care, General practice, Complex intervention, Older people, Process evaluation

## Abstract

**Background:**

The PolyPrime intervention is a theory-based intervention aimed at improving appropriate polypharmacy in older people (aged ≥65 years) in primary care. The intervention consists of an online video which demonstrates how general practitioners (GPs) can prescribe appropriate polypharmacy during a consultation with an older patient and a patient recall process, whereby patients are invited to scheduled medication review consultations with GPs. The aim of the process evaluation is to further examine the implementation of the PolyPrime intervention in primary care. This will involve investigating whether the PolyPrime intervention can be delivered as intended across two healthcare systems, how acceptable the intervention is to GPs, practice staff and patients, and to identify the intervention’s likely mechanisms of action.

**Methods:**

The PolyPrime study is an external pilot cluster randomised controlled trial (cRCT) which aims to recruit 12 GP practices across Northern Ireland [NI] (n=6) and the six counties in the Republic of Ireland (ROI) that border NI (*n*=6). Practices have been randomised to intervention or usual care. An embedded process evaluation will assess intervention fidelity (i.e. was the intervention delivered as intended), acceptability of the intervention to GPs, practice staff and patients and potential mechanisms of action (i.e. what components of the intervention were perceived to be effective). Quantitative data will be collected from data collection forms completed by GPs and practice staff and a feedback questionnaire completed by patients from intervention arm practices, which will be analysed using descriptive statistics. Qualitative data will be collected through semi-structured interviews with GPs and practice staff and audio-recordings of medication review appointments from the intervention arm practices which will be transcribed and analysed using the framework method. Quantitative and qualitative data will be triangulated to provide an overall assessment of intervention fidelity, intervention acceptability, and mechanisms of action.

**Discussion:**

This process evaluation will add to feasibility data from the pilot cRCT by providing evidence on the fidelity of implementing the intervention package across two healthcare systems, the acceptability of the intervention and potential mechanisms of action.

**Trial registration:**

ClinicalTrials.govISRCTN41009897. Registered on 19 November 2019. ClinicalTrials.govNCT04181879. Registered 02 December 2019.

**Supplementary Information:**

The online version contains supplementary material available at 10.1186/s13063-021-05410-6.

## Background

The use of multiple medications (polypharmacy) is increasingly seen as ‘potentially problematic rather than always inappropriate’ [1]. However, the growing prevalence of multimorbidity often drives prescribing, which is based on multiple guidelines [2]. As such, assessments of prescribing appropriateness should extend beyond the number of medications prescribed and differentiate between ‘many’ medicines (appropriate polypharmacy) and ‘too many’ medicines (inappropriate polypharmacy) [1]. Several papers have outlined the need for a systematic approach to tackling polypharmacy, particularly in primary care [3–5]. A Cochrane systematic review, which evaluated interventions to improve the appropriate prescribing of polypharmacy for older people [6] highlighted several deficits in previous intervention studies. For example, the methods sections of included studies did not describe the development of the interventions, whether evidence-based techniques for changing behaviour had been considered, and whether healthcare professionals (HCPs) contributed to the development of the interventions [6]. It is now recognised that more time should be spent on intervention development, using a systematic approach that incorporates a sound theoretical basis and involves those who deliver and/or receive these interventions, i.e. healthcare professionals, patient and carers [7]. Such an approach has been advocated by the United Kingdom’s Medical Research Council (MRC) guidance on complex interventions [8, 9].

### The PolyPrime intervention

The PolyPrime intervention is a theory-based intervention aimed at improving appropriate polypharmacy in older people (aged ≥65 years) in primary care and was developed in accordance with the MRC guidance [10–12]. This involved conducting Theoretical Domains Framework (TDF)-based semi-structured interviews with general practitioners (GPs) and community pharmacists [10]. The TDF is an integrated framework of behaviour change theories that enables mediators of behaviour change to be identified and targeted. These interviews aimed to identify potential barriers and facilitators to prescribing and dispensing appropriate polypharmacy, which were then mapped to four behaviour change techniques (BCTs) to form the “active components” of the intervention [11]. The intervention, which was then tested in a small-scale feasibility study [12], consists of two main components: an online video demonstrating how GPs can improve appropriate polypharmacy during typical consultations with older patients (BCT: ‘Modelling or demonstrating of behaviour’; [13]) and a patient recall process, whereby patients are invited to medication review consultations with GPs (on two occasions; an initial medication review and a 6-month follow-up appointment with the same GP) [12, 14]. The video includes feedback from both a practising GP and a simulated patient emphasising the positive outcomes of the consultation (BCT: ‘Salience of consequences’ [13]). The video also includes additional educational slides highlighting key issues which GPs should consider when conducting the medication reviews, along with links to additional resources for GPs [14, 15]. To facilitate the patient recall process, two complementary intervention components involve GPs making explicit plans at weekly practice staff meetings of when and how they would ensure that target patients are prescribed appropriate polypharmacy (BCT: ‘Action planning’ [13]) and GPs receiving prompts from reception staff to carry out this plan when target patients attend their appointments (BCT: ‘Prompts/cues’ [13]).

### Process evaluations

The MRC guidance also states that process evaluation is an important step in developing and testing complex interventions [8, 9]. Process evaluations are studies that run parallel to, or follow, intervention trials and which can be used to assess intervention implementation, potential mechanism of impact, and to identify relevant contextual factors (i.e. barriers or facilitators to the implementation or effects of the intervention) [9]. These evaluations align with the underlying aims of pilot studies which seek to evaluate the feasibility of recruitment, retention, assessment procedures, and implementation of a novel intervention, which often include assessments of intervention fidelity [16].

Investigating how complex interventions, which often include multiple components, are implemented is imperative in understanding how each component works in practice and if modifications are needed before proceeding to larger scale trials [17]. One of the main aspects of intervention implementation is intervention fidelity, defined as whether the intervention is delivered as intended [9]. The Treatment Fidelity Workgroup of the NIH Behavioral Change Consortium has recommended that the following five areas need to be addressed to assess intervention fidelity: study design, training, treatment delivery, treatment receipt, and treatment enactment [18, 19]. It is also assumed that when an intervention or the ‘active components’ of the intervention are delivered as per protocol and a high level of fidelity is achieved, researchers can have greater confidence in the research outcomes [20].

The successful implementation of a complex intervention often depends on the acceptability of the intervention to both those delivering and receiving the intervention [21]. While the MRC guidance highlights that assessing acceptability is an important element within a process evaluation study, they do not provide guidance on the specific methodology to achieve this [9, 22]. Sekhon et al. who define acceptability as *a multi-faceted construct that reflects the extent to which people delivering or receiving a healthcare intervention consider it to be appropriate, based on anticipated or experienced cognitive and emotional responses to the intervention* have developed a theoretical framework of acceptability (TFA). The TFA consists of seven constructs: affective attitude, burden, intervention coherence, ethicality, opportunity costs, perceived effectiveness and self-efficacy, which researchers can use to guide assessments of acceptability (see Table 1) [22, 23]. Previous research has found that the use of this framework provided a more substantial assessment of intervention acceptability when asking participants about this construct [24].

**Table 1:** Definitions of the component constructs in the theoretical framework of acceptability (TFA) applied to PolyPrime

TFA construct	Definition	GPs	Practice Staff	Patients
Affective attitude	How an individual feels about the intervention	✔	✔	✔
Burden	The perceived amount of effort that is required to participate in the intervention	✔	✔	✔
Ethicality	The extent to which the intervention has good fit with an individual’s value system			
Intervention coherence	The extent to which the participant understands the intervention and how it works	✔		
Opportunity costs	The extent to which benefits, profits, or values must be given up to engage in the intervention	✔	✔	
Perceived effectiveness	The extent to which the intervention is perceived to be likely to achieve its purpose	✔		✔
Self-efficacy	The participant’s confidence that they can perform the behaviour(s) required to participate in the intervention	✔		

Adapted from [22]

The MRC guidance also suggests that exploring the potential mechanisms of action (i.e. the process by which an intervention component exerts its effect) is important in understanding how the intervention works and which components are essential. The use of a mixed methods approach is encouraged, whereby quantitative data can be used to investigate the hypothesised causal pathways, and qualitative data can be used to better understand the pathways from the participants’ perspective [25]. This often utilises the theory underlying intervention development to focus the evaluation and provide additional insight into causal mechanisms affecting outcomes [26].

This paper describes the rationale, methods and analysis plan for an embedded process evaluation for the PolyPrime intervention. This external pilot cluster randomised controlled trial is ongoing and will be completed by January 2022. A protocol paper for the main pilot trial has been published [14].

## Methods/design

### Aim

The aim of the PolyPrime process evaluation is to test the implementation of the PolyPrime intervention in primary care.

The objectives of the study are to:

1. Assess if the intervention is delivered and received as intended (intervention fidelity)
2. Assess the acceptability of the intervention to GPs, practice staff and patients
3. Identify the intervention’s likely mechanisms of action.

## Methods

### Study design

A mixed methods process evaluation study will be undertaken, involving interviews with GPs and practice staff, patient feedback questionnaires, data collection forms completed by practice staff and audio-recordings of patient medication review appointments. Ethical approval has been obtained from by the North of Scotland (REC reference: 19/NS/0100) and the Irish College of General Practitioners Research Ethics Committees (RECs).

### Setting

**Fig. 1:**
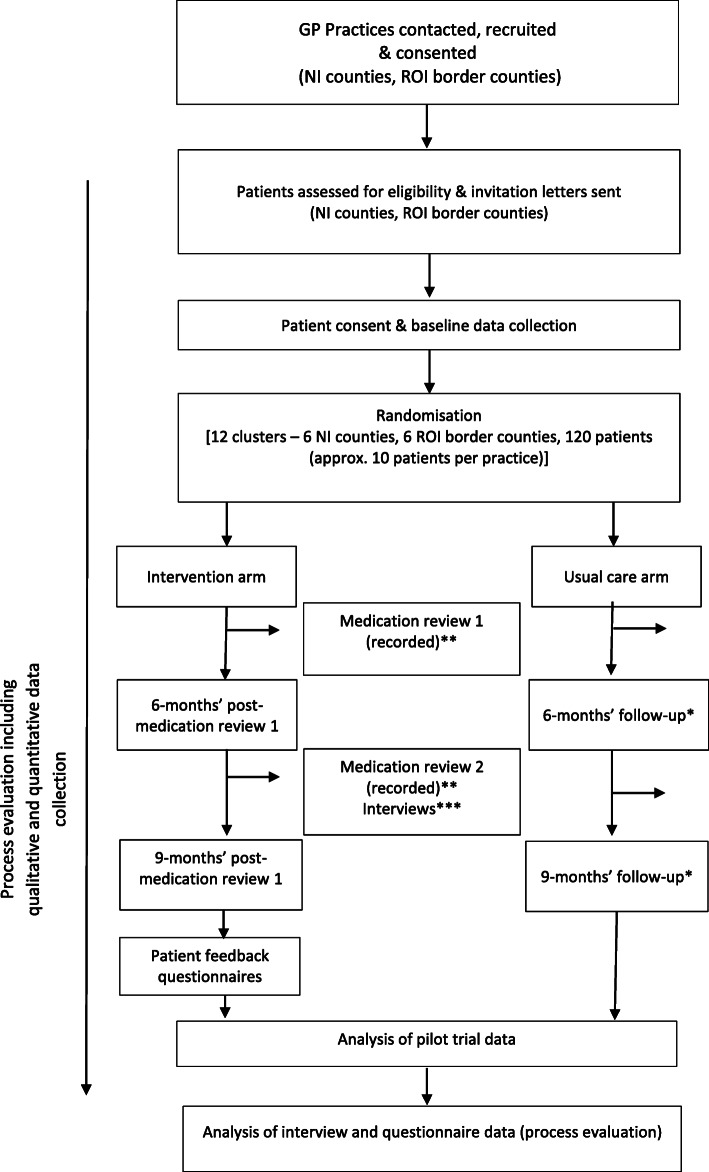
Overview of the PolyPrime study and process evaluation activities. *The follow-up time points for the control arm will be based on the average length of time from the completion of baseline data collection to 6 and 9 months post initial medication review in the intervention arm. **Process evaluation activities—counts of GP viewing online video, weekly practice meetings to discuss medication reviews, scheduling of appointments and prompts given to GPs by practice staff to conduct medication reviews. ***Process evaluation activities—semi-structured interviews with GPs and practice staff after medication review 2. NI-Northern Ireland; ROI Republic of Ireland

The main PolyPrime study is currently being conducted in nine GP practices in Northern Ireland (NI) and the border counties of the Republic of Ireland [ROI] (Donegal, Leitrim, Sligo, Cavan, Monaghan and Louth). Due to the COVID-19 pandemic, three practices have withdrawn from the original 12 which were initially recruited. The process evaluation will be conducted only in the GP practices randomised to the intervention arm. An overview of the main pilot trial and process evaluation activities can be seen in Fig. 1.

### GP and practice staff recruitment

A detailed overview of the sampling and recruitment procedures for GPs and patients has been described in detail elsewhere [14]. Briefly, GP practice recruitment (now complete) took place in two stages. Firstly, a random sample of GP practices was contacted via a letter seeking expressions of interest. The second stage involved research nurses from the Northern Ireland Clinical Research Network (NICRN – Primary Care) and Trinity College Dublin (TCD) telephoning the practice manager or lead GP in each practice to determine their interest in receiving information about the study. GPs were asked to provide written informed consent, which included agreeing to take part in an interview towards the end of the study and to audio-record the discussion during a medication review with a patient’s consent should the practice be randomised to the intervention arm.

One member of practice staff (i.e. practice manager or receptionist) from each intervention arm practice (i.e. those involved in implementing the intervention within the practice) was recruited to facilitate implementation of the intervention (i.e. patient recruitment and scheduling medication review appointments), complete data collection forms and to participate in a post-intervention feedback interview. Practice staff received an information sheet providing details about the interviews and were asked to provide written informed consent.

### Patient recruitment

Following GP practice recruitment, practice staff were asked to screen patient records to identify potentially eligible patients with the goal of recruiting up to 10 patients per practice (for details on patient recruitment, see protocol paper for the main pilot trial [14]). Patients have been asked to provide written informed consent, which includes agreeing to complete a feedback questionnaire towards the end of the study and to the GP audio-recording the discussion during their medication review appointments should they be from a GP practice randomised to the intervention arm. Patients will still be able to participate in the main pilot cRCT even if they do not agree to have their medication review appointments audio-recorded. At the time of submission, patient recruitment is almost complete.

### Data collection

#### Intervention fidelity

Data will be collected on intervention fidelity [9] as summarised in Table 2; this will be evaluated in relation to the delivery, receipt and/or enactment of the four intervention components (i.e. online video, patient recall, weekly meetings and prompts/cues). Firstly, delivery and/or receipt of each of the four intervention components by GPs will be explored. The practice staff role in facilitating the implementation of the prompts/cues component of the intervention will also be examined. Secondly, enactment of the intervention (by GPs) will be explored (i.e. whether medication reviews were completed by GPs as intended). Finally, the delivery of any additional BCTs aimed at improving the prescribing of appropriate polypharmacy during the medication reviews with patients by GPs will be assessed (see Table 2).

**Table 2:** Intervention fidelity data collected as part of process evaluation

Intervention component	Aim (what is being assessed)	Data collected	Data source^**a**^
Online video	Delivery/receipt of the intervention	The number of times the GPs access the online video	Online video administration page
Weekly meetings	Delivery/receipt of the intervention	The number of weekly practice staff meetings held (at which explicit plans will be made to recall patients for medication reviews)	Data collection forms completed by practice staff
Prompts/cues	Delivery/receipt of the intervention	The number of prompts GPs receive from practice staff to conduct medication reviews	Data collection forms completed by practice staff
Patient recall	Delivery/receipt of the intervention	The number of appointments scheduled and attended	Data collection forms completed by practice staff
	Enactment of the intervention	Fidelity of medication reviews conducted by GPs Additional BCTs delivered to the patient by the GP during the process of the medication review	Audio-recorded medication reviews

*BCT* behaviour change technique, *GP* General Practitioner

^a^Based on data collected from the 6 intervention arm GP practices

#### Intervention acceptability

Data will be collected on the acceptability of study procedures and the acceptability of the intervention to GPs, practice staff and patients (see the “Data collection methods” section). Semi-structured interview and questionnaire items relating to the acceptability of the overall intervention have been framed using the constructs of the TFA: affective attitude, burden, intervention coherence, opportunity costs, perceived effectiveness and self-efficacy (Table 1) [22]. However, questions relating to the TFA constructs ‘self-efficacy’, ‘opportunity costs’ or ‘intervention coherence’ have not been included in the patient questionnaire as the intervention is aiming to change what GPs do as part of delivering routine care, rather than what patients do. Similarly, questions relating to the constructs ‘self-efficacy’, ‘perceived effectiveness’ or ‘intervention coherence’ have not been included in the practice staff interview topic guide. Finally, due to the nature of the intervention and following consultation with the various stakeholders on the Project Management Team, a question relating to the TFA construct ‘ethicality’ (i.e. the extent to which the intervention was a good fit with an individual’s value system) has not been included. GPs, practice staff and patients will also be asked a broad ‘yes or no’ question regarding the acceptability of the overall intervention.

#### Mechanisms of action

Data will be collected to identify the intervention’s likely mechanisms of action (i.e. the process whereby an intervention component exerts its effect). Firstly, we will explore if the online video component brought about changes in the GPs’ beliefs about capabilities (e.g. their self-efficacy) [27] to provide appropriate polypharmacy for the effective clinical management of older people in primary care. Secondly, we will explore if the patient recall process was effective in bringing about positive attitudes towards the medication reviews (‘Salience of consequences’ for GPs). Finally, we will explore if weekly meetings (BCT: ‘Action planning’) and practice staff delivering prompts to GPs (BCT: ‘Prompts/cues’) were perceived to be effective and/or useful in reminding GP practices to organise appointments and reminding GPs to deliver a medication review to the patient (see Table 3). In our qualitative assessment of the likely mechanisms of action for the PolyPrime intervention, we will use established methods that link BCTs to theoretically defined mechanisms of action [28]. For example, we can assess the extent to which the BCT ‘prompts/cues’ was perceived to influence memory, attention and decision processes or relevant behavioural cueing processes that help initiate and maintain medication reviews, as part of routine care. This analysis of potential mechanisms of action will help refine the process evaluation and theoretical underpinning of any subsequent full trial of future iterations of the PolyPrime intervention [9]. This will be integrated into the interpretation phase of the framework analyses described below.

**Table 3:** Intervention component and data collected as part of process evaluation

Intervention component	Impact	Data collected	Data source^**a**^
Online video	Changes in the GPs’ beliefs about capabilities (e.g. their self-efficacy) to prescribe appropriate polypharmacy during a consultation with an older patient	GP views on the usefulness of the online video	GP feedback interviews
Weekly meetings	Weekly meetings are perceived to be effective in GPs making plans to schedule patient appointments	The number of weekly practice staff meetings held	Data collection forms completed by practice staff
		GP views on the effectiveness/usefulness of weekly meetings	GP feedback interviews
		Practice staff views on the effectiveness/usefulness of weekly meetings	Practice staff feedback interviews
Prompts/cues	Prompts from practice staff were perceived to be effective in reminding GPs to deliver a medication review to patients	The number of prompts GPs receive from practice staff to conduct medication reviews	Data collection forms completed by practice staff
		GP views on the effectiveness/usefulness of receiving prompts	GP feedback interviews
		Practice staff views on the effectiveness/usefulness of delivering prompts	Practice staff feedback interviews
Patient recall	GPs reporting positive attitudes towards the medication review	GP views on the impact of the intervention	GP feedback interviews

*GP* General Practitioner

^a^Based on data collected from the 6 intervention arm GP practices

### Data collection methods

The following text refers to the data sources identified in Tables 2 and 3.

### Quantitative data

The following data will be collected on study-specific data collection forms:

- *The number of times the GPs (allocated to the intervention arm) access the online video:* In order to collect information on the number of times each GP accessed the online server to watch the video, an administrator login page has been developed which can be accessed by the research team. This page will record the following details for each GP (based on their individual usernames): number of times logged in, date of last login and number of times pressed play (on the video).
- *The number of weekly practice staff meetings held (at which explicit plans will be made to recall patients for medication reviews):* Practice staff will be asked to record the number of weekly practice staff meetings held in the intervention arm practices, and during which, plans are made to recall patients for medication review in the form of a scheduled appointment.
- *The number of prompts GPs receive from practice staff to conduct medication reviews:* Practice staff will be asked to record the number of prompts GPs receive from practice staff to conduct medication reviews. Practice staff will record details on which member of practice staff delivered the prompt, the number of prompts made to the GP and how prompts were given (i.e. verbally or using a computerised system). Practice staff will be asked to provide this information for both the initial and 6-month follow-up medication review appointments.
- *Patient feedback questionnaire:* Patients from the intervention arm practices will be asked to complete a feedback questionnaire (see Additional File 1) as soon as possible after delivery of the intervention (i.e. after completion of their final follow-up questionnaires). The questionnaires will be posted to patients by the Research Fellow/Assistant, and they will be asked to return them using a pre-paid envelope provided. Patients will be telephoned as a reminder to complete the feedback questionnaire if this has not been returned, and where necessary, patients will be given the opportunity to complete this via the telephone. Patients will be asked about their views on the following:
  Acceptability of study procedures including recruitment, completing questionnaires and support provided by the research team
  Medication review appointments—including mode of delivery
  Acceptability of the intervention (questions based on TFA) (Table 1) [22].

### Qualitative data

#### GP feedback interview

At the end of the intervention delivery phase (i.e. after both the initial and 6-month medication reviews are completed), qualitative interviews will be conducted with up to 10 GPs in the intervention arm practices. GPs will be interviewed as soon as possible after receipt of the training to deliver the PolyPrime intervention and subsequent delivery of the intervention to patients, using a semi-structured interview guide. The interviews will be completed either face-to-face at a location that is mutually convenient for both the GPs and the researcher (e.g. GP practices), via the telephone or video-call. GPs will be given an honorarium of £46/€54 to compensate them for the time associated with participation in an interview. GPs will be asked about their views on the following:

- Acceptability of study procedures including patient screening and recruitment and support provided by the research team
- Experience of the PolyPrime intervention including how it enhanced knowledge, skills and confidence in abilities
  - Online video (and supporting materials)
  - Weekly practice meetings
  - Prompts/cues
  - Patient recall process—including mode of delivery
- Acceptability of the intervention (questions based on TFA) (Table 1) [22].

#### Practice staff feedback interview

At the end of the intervention delivery phase (i.e. after both the initial and 6-month medication reviews are completed), an interview will be conducted with one member of practice staff (i.e. practice manager or receptionist) from each intervention arm practice (i.e. those involved in implementing the intervention within the practice). Practice staff will be interviewed via telephone as soon as possible after medication reviews have been completed with all recruited patients, using a semi-structured interview guide, at a time convenient to them. Practice staff will be asked about their views on the following:

- Acceptability of study procedures including patient screening and recruitment and support provided by the research team
- Experience of the PolyPrime intervention
  - Weekly practice meetings
  - Scheduling appointments
  - Prompts/cues
- Acceptability of the intervention (questions based on TFA) (Table 1) [22].

#### Audio-recorded medication reviews

As outlined above (see GP and patient recruitment section), GPs and patients will be asked to consent to having the medication review discussions audio-recorded. Due to the current coronavirus restrictions in place, these medication reviews will be conducted either face-to-face, via the telephone or video-call. If possible, each recruited GP from the intervention arm practices will be asked to audio-record one patient’s initial and 6-month follow-up medication review appointments. The GPs will be reminded by a member of the research team to undertake this activity for one of their recruited patients. Audio-recording equipment will be provided by the research team.

### Quantitative analysis

Analysis in relation to study-specific data collection forms and patient questionnaires will use descriptive statistics (e.g. counts, means, standard deviations) and will be conducted using SPSS (version 26.0). Recorded practice-level data will relate to the number of times the intervention GPs access the online video, the number of weekly practice staff meetings held at which explicit plans are made to recall patients for medication reviews, and the number of prompts GPs receive from practice staff to conduct medication reviews. These counts will provide an indication as to whether the intervention has been delivered and received as intended (i.e. intervention fidelity), in conjunction with the outcome data collected in the pilot trial [14]. Patient-level data will be available from the patient feedback questionnaires (see Additional File 1). The acceptability of the study procedures and medication reviews to patients will be assessed via responses to questions (yes/no), scales (Likert-type) or a series of statements relating to acceptability.

### Qualitative analysis—feedback interviews

Analysis of the post-intervention feedback interviews will use a framework analysis [29, 30], which will involve seven stages. The first stage will involve an in-depth familiarisation process. Coding of the transcripts will then take place in two phases. The second stage (first coding phase) will adopt a deductive approach using the TFA constructs [22] to frame the questions relating to the overall acceptability of the intervention in the GP and practice staff interviews (see the “Intervention acceptability” section and Table 1). The third stage (second coding phase) will adopt an inductive approach, in which emerging themes will be identified. This will be followed by the development of a working analytical framework (Stage 4), applying the analytical framework (Stage 5), charting data into the framework matrix (Stage 6) and interpreting the data (Stage 7). All transcripts will be analysed independently by at least two members of the research team. Two sets of analysis will be undertaken, one for each professional group (i.e. GPs and practice staff).

### Qualitative data analysis—medication review appointments

Analysis of the medication review appointment transcripts will be conducted in a similar way to that outlined above. The second stage (first coding phase) will adopt a deductive approach using the BCT taxonomy [13] which will be used to assess if GPs delivered any additional BCTs during the patient recall process. The third stage (second coding phase) will adopt an inductive approach, in which any additional emerging themes will be identified.

### Triangulation of quantitative and qualitative data

Once all quantitative and qualitative data have been analysed separately by the researchers, the results from all five data sets (GP feedback interviews, practice staff interviews, patient feedback questionnaires, medication review appointment recordings, study-specific data collection forms) will be compared. This will follow a triangulation protocol [31] involving six steps (Table 4). Step 1 will involve two researchers examining the interpretative summaries of all analyses to identify the key findings for each data set. The findings related to each research question (outlined above) from all five data sets will be sorted and separated into three files (one for each research question—fidelity, acceptability, mechanisms of action). Step 2 will involve researchers reviewing the contents of each file to identify the key themes discussed in each data set to create a unified list of themes. These themes will form the rows of a convergence coding matrix used to summarise similarities from the second step; each key finding will be compared across the five data sets to create the matrix. For each key finding, paired comparisons will be made to compare the data coming from each data set. The relationship between data will be marked as one of four categories: silence, dissonance (or disagreement), partial agreement and agreement (see Table 4). Comparisons will be labelled as not applicable to denote the source of data which has supplied a theme or when neither data set in a paired comparison contained data related to the finding. This will be followed by convergence assessment (Step 3) and completeness comparison (Step 4). The level of agreement between the two researchers with respect to the degree of convergence between data sets (in both meaning and prominence of key findings) will then be calculated (Step 5). A method established by Miles and Huberman will be used, whereby agreements are scored with 1 and disagreements scored with 0, and the percentage agreement calculated [32]. Miles and Huberman suggest that an inter-coder reliability of 80% agreement on level of convergence is acceptable agreement among multiple coders. Any disagreements will be resolved by consensus through discussion with another researcher. The triangulated results will then be presented to the wider research team for discussion and to make a final decision on the interpretation of the findings (Step 6). All process evaluation activities have been summarised in Table 5.

**Table 4:** Triangulation protocol

Step	Activity
1. Sorting	Sort findings from each data set to identify the key findings that address each research question of interest to determine areas of content overlap and divergence. A list of the key themes will be compiled and added to the convergence coding matrix
2. Convergence coding	Compare the findings to determine the degree of convergence of (a) essence of the meaning and prominence (e.g. the number of participants mentioning a theme) of the themes presented and (b) specific examples provided in relation to each theme. Characterise the degree and type of convergence using the following concurrence coding scheme within theme areas:
Convergence coding scheme	
Agreement	There is full agreement between the sets of results on both elements of comparison (e.g. meaning and prominence are the same and specific examples provided are the same)
Partial agreement	There is agreement on one but not both components (e.g. the meaning or prominence of themes is the same or specific examples provided are the same)
Silence	One set of results covers the theme or example, whereas the other set of results is silent on the theme or example
Dissonance	There is disagreement between the sets of results on both elements of comparison (e.g. meaning and prominence are different and specific examples provided are different)
3. Convergence assessment	Review convergence coding matrix to provide a global assessment of the level of convergence. Document when and where researchers have different perspectives on convergence or dissonance of findings
4. Completeness comparison	Compare the findings from the convergence coding matrix to create an overarching summary of the findings, highlighting both unique and similar contributions to each research question
5. Researcher comparison	Determine the degree of agreement among researchers on triangulated findings. Any disagreements will be resolved by consensus through discussion with another researcher
6. Feedback	Feedback of triangulated results to the wider research team for review and clarification

Adapted from [31]

**Table 5:** Process evaluation overview

Activity	Data collection	Participants	Timepoint	Duration	Analysis
Online video access	No. of times logged in dates, no. of times pressed play	GPs^a^	Before medication reviews 1 and 2^b^	n/a	Counts
Weekly practice meetings	No. of meetings	Practice staff^a^	Before medication reviews 1 and 2^b^	n/a	Counts
Medication review appointments	No. of appointments scheduled and attended	Practice staff^a^	Before and medication reviews 1 and 2^b^	n/a	Counts and reasons for non-attendance
Prompts	No. of prompts GPs receive from practice staff to conduct medication reviews	GPs and practice staff^a^	Before and medication reviews 1 and 2^b^	n/a	Counts
Medication review	Audio-recording	GPs^a^	Medication reviews 1 and 2^b^	10-30 mins	Thematic analysis; BCT coding
GP feedback	Semi-structured interview	GPs^a^ (one per practice)	After medication review 2	Up to 45 mins	Thematic analysis
Practice staff feedback	Semi-structured interview	Practice staff^a^ (one per practice)	After medication review 2	Up to 30 mins	Thematic analysis
Patient feedback	Questionnaire	Patients^a^ (all)	After 9-month follow-up questionnaires completed	10-15 mins	Quantitative and qualitative

^a^From intervention arm practices

^b^Initial medication review (1) and six-month follow-up medication review (2)

### Data management and monitoring

All participants (patients, GPs, practice staff and GP practices) will be given a unique study ID number and data will be anonymised/pseudonymised (e.g. study-specific data collection forms and interview transcripts). Personal data including consent forms, completed questionnaires or transcripts will be held in a locked filing cabinet, within a locked office on a secure keycode-protected floor of the School of Pharmacy, Queen’s University Belfast (QUB) or the School of Pharmacy and Pharmaceutical Sciences, TCD. Electronic data will be stored within the QUB or TCD network space and will be password-protected to ensure confidentiality. Interviews will be digitally recorded (with permission and written, informed consent), transcribed and checked for accuracy. Once transcripts have been checked for accuracy recordings will be deleted and transcriptions stored within QUB or TCD. All participant consent forms, questionnaires, study-specific data collection forms and transcripts stored at TCD will be transferred to QUB, in line with General Data Protection Regulation guidelines for transferral of data, upon study completion. This will be done via recorded delivery for hard copy data and encrypted email for electronic data. When the study has been completed, the forms and transcripts will be securely stored for 5 years and then destroyed. The investigators will conduct the study in compliance with the protocol given approval/favourable opinion by the relevant RECs. Changes to the protocol may require REC approval prior to implementation, except when modification is needed to eliminate an immediate hazard(s) to patients. The CI and trial team, in collaboration with the sponsor, will submit all protocol modifications to the RECs for review in accordance with the governing regulations. Protocol compliance will be monitored by the trial team who will undertake site visits or telephone calls (if pandemic restrictions are in place) to ensure that the trial protocol is adhered to, such as, scheduling of medication reviews, contacting patients for appointments). Study paperwork will be reviewed, such as questionnaires and consent forms, to ensure that they are being completed appropriately and fully. An independent Trial Steering Committee has been established (consisting of two academic GPs, a health services researcher and statistician) which is overseeing the conduct of the trial and advising on safety in respect of the study.

The findings of this process evaluation will be communicated to all participants, published in relevant journals and presented at conferences. In addition to publishing scientific papers, we also plan to host a seminar for participating practices and patients in convenient locations to present the findings to them. This will be contingent on any pandemic restrictions that may be in place at that time.

## Discussion

The process evaluation will explore whether the PolyPrime intervention is delivered as intended, if the various study procedures are acceptable for GPs, practice staff and patients and potential mechanisms of action. This will add to the data from the main external pilot cRCT of the PolyPrime intervention which aims to provide information about the feasibility of recruitment, retention and study procedures, including collecting data on medication appropriateness (from GP records), quality of life and health service use (i.e. hospitalisations) [14].

Embedding the process evaluation to run alongside the pilot study will enable the collection of data in real-time. This is particularly important in multi-site cRCTs to investigate if the intervention components are delivered in the same way across sites [17]. This is of significance in the PolyPrime intervention given the cross-border nature of the study, where it will be essential to understand how feasible the intervention is to deliver in both healthcare settings. Furthermore, understanding if study procedures or intervention components are feasible and acceptable to those delivering and/or receiving the intervention is important to consider before moving forward to a larger definitive trial, while modifications can be made.

The use of both qualitative and quantitative methods in the process evaluation will allow a detailed evaluation of intervention implementation in each GP practice. Quantitative data relating to the delivery, receipt and/or enactment of each intervention component will give an indication of whether these can be integrated into general practice. The interview data regarding GPs’ and practice staffs’ experiences of implementing the PolyPrime intervention in practice will provide in-depth insight into those processes. Furthermore, the use of feedback questionnaires with patients regarding their experiences of being involved in the PolyPrime intervention will provide an evaluation of the acceptability of study procedures and highlight areas for potential refinement before attempting to recruit a larger number of patients into a future definitive trial. Additionally, the use of TFA constructs [24] to guide the development of the patient feedback questionnaire and GP and practice staff interview topic guide will also provide a substantial assessment of intervention acceptability. Finally, both quantitative and qualitative data will be used to highlight which components of the intervention are likely to influence GP prescribing behaviour and the potential mechanisms of action that are likely to account for any observed changes.

### Trial status

This study was registered at ISRCTN (10.1186/ISRCTN41009897) on 19 November 2019 and ClinicalTrials.gov (https://clinicaltrials.gov/ct2/show/NCT04181879) on 02 December 2019. At the time of resubmission of the revised manuscript (NI protocol version 4.0; date, 20 November 2020; ROI protocol version 5.0; date, 30 March 2021), 9 GP practices had been retained in the study following three drop-outs due to logistical issues arising from the COVID-19 pandemic, and 58 patients have been recruited and retained in the study.

## Supplementary Information

**Additional file 1.** Patient feedback questionnaire. Questionnaire to be administered to patients from intervention arm practices following delivery of the intervention.

## Data Availability

The datasets used and/or analysed during the current study will be available from the corresponding author on reasonable request.

## Abbreviations

cRCT: Cluster randomised controlled trial
BCT: Behavioural change technique
GP: General practitioner
HCP: Healthcare professional
MRC: Medical Research Council
NHS: National Health Service
NI: Northern Ireland
NICRN: Northern Ireland Clinical Research Network
QUB: Queen’s University Belfast
RCT: Randomised controlled trial
REC: Research Ethics Committee
ROI: Republic of Ireland
TCD: Trinity College Dublin
TDF: Theoretical Domains Framework
TFA: Theoretical framework of acceptability
