# Supplementary material for: Protocol for a process evaluation of an external pilot cluster randomised controlled trial of a theory-based intervention to improve appropriate polypharmacy in older people in primary care: the PolyPrime study
Source: Trials. 2021 Jul 14;22:449. doi: 10.1186/s13063-021-05410-6 (PMC8278187; doi:10.1186/s13063-021-05410-6)
Supplement: Supplementary file 1 — Additional file 1. Patient feedback questionnaire. Questionnaire to be administered to patients from intervention arm practices following delivery of the intervention. [file 13063_2021_5410_MOESM1_ESM.docx]

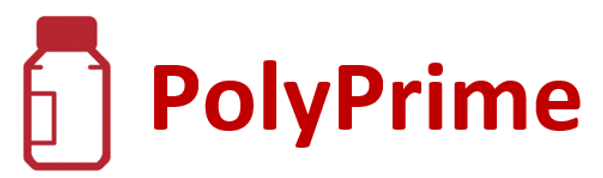

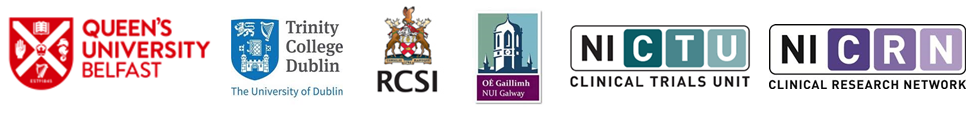


**Patient feedback questionnaire**

We would like to hear your thoughts about the PolyPrime study that you took part in, so that we can continue to improve our research, and help support people who take many medicines. We want to know your honest thoughts about the study, and we would welcome any feedback that you may have. We have developed a short questionnaire that asks for your views about the study, the medication reviews that you received from your general practitioner (GP), and your overall experience of being involved in the PolyPrime study.

Once you have completed the questionnaire, you can use the return envelope provided to send it straight back to a member of the research team.

**Your GP will not see your answers to these questions**.

If you have any questions about this questionnaire, you can get in touch using the contact details below.

| If you live in **Northern Ireland** | If you live in the **Republic of Ireland** |
| --- | --- |
| Dr. Audrey Rankin | Ms. Ashleigh Gorman |
| Research Fellow | Research Assistant |
| School of Pharmacy | School of Pharmacy and Pharmaceutical Sciences |
| Queen's University Belfast | Trinity College Dublin |
| 97 Lisburn Road | Panoz Institute |
| Belfast BT9 7BL | Dublin D02PN40 |
| Telephone: +44 (0) 7391 730647 | Telephone: +353 (0) 86 608 9094 |
| Email: a.rankin@qub.ac.uk | Email: gormanas@tcd.ie |
|  |  |

| **Part 1 – Study procedures** | | | | | |
| --- | --- | --- | --- | --- | --- |
| 1. **Think about the first time you were contacted about this study through the post. Did you like or dislike the way you were contacted? *Please circle one of the following:*** | | | | | |
| Strongly like | Like | No opinion | Dislike | Strongly dislike | |
| 1. **If you circled ‘Dislike’ or ‘Strongly dislike’ to Question 1, please briefly explain your reasons for doing this:** | | | | | |
| 1. **If you circled ‘Dislike’ or ‘Strongly dislike’ to Question 1, what would have been a better way to contact you about getting involved in this study?** | | | | | |
| 1. **During the study you were asked to complete questionnaires on three occasions about your quality of life and how you used health services. Were you happy with the number of questionnaires you were asked to complete during the study? *Please tick the appropriate box:*** | | | | | |
| Yes | | | | |  |
| No | | | | |  |
| 1. **Please briefly explain your reasons for stating this:** | | | | | |
| 1. **Were you happy with the support provided by members of the research team (i.e. the members of the research team listed on page 1)? *Please tick the appropriate box:*** | | | | | |
| Yes | | | | |  |
| No | | | | |  |
| 1. **Please briefly explain your reasons for stating this:** | | | | | |

| **Part 2 – Your medication reviews during the PolyPrime study** | | | | | |
| --- | --- | --- | --- | --- | --- |
| 1. **What did you hope would happen as a result of having your medicines reviewed by your GP?** ***Please tick all that apply:*** | | | | | |
| The number of medicines I take would decrease | | | | |  |
| The number of medicines I take would increase | | | | |  |
| The number of times I take my medicines each day would decrease | | | | |  |
| The number of times I take my medicines each day would increase | | | | |  |
| I would have a better understanding about the medicines I take | | | | |  |
| I would feel happier about my medicines I take | | | | |  |
| I would feel reassured that my medicines have been reviewed | | | | |  |
| Nothing, please briefly explain why: | | | | |  |
| If you thought something else would happen, please briefly explain: | | | | |  |
| **In the following questions, we would like you to think about the first medication review appointment you received as part of the PolyPrime study.** | | | | | |
| 1. **How did your first medication review take place? *Please tick the appropriate box:*** | | | | | |
| During a face-to-face appointment | | | | |  |
| By telephone | | | | |  |
| By video call | | | | |  |
| 1. **Did you like or dislike the way you received your first medication review (i.e. face-to-face, over the telephone or video call)? *Please circle one of the following:*** | | | | | |
| Strongly like | Like | No opinion | Dislike | Strongly dislike | |
| 1. **Please briefly explain your reasons for stating this:** | | | | | |
| 1. **Did the doctor recommend any changes to the medicines that you were taking at the time of the first medication review? *Please tick the appropriate box:*** | | | | | |
| Yes (If YES to Question 12, please complete Questions 13 & 14) | | | | |  |
| No (If NO to Question 12, please go to Question 15) | | | | |  |
| 1. **Did you agree with the doctor’s recommended change(s) to the medicines that you were taking at the time of the first review? *Please tick the appropriate box:*** | | | | | |
| Yes | | | | |  |
| No | | | | |  |
| 1. **Please briefly explain your reasons for agreeing/not agreeing with the change(s) that the doctor recommended:** | | | | | |
| **In the following questions we would like you to think about the second medication review appointment you received as part of the PolyPrime study.** | | | | | |
| 1. **How did your second medication review take place? *Please tick the appropriate box:*** | | | | | |
| During a face-to-face appointment | | | | |  |
| By telephone | | | | |  |
| By video call | | | | |  |
| 1. **Did you like or dislike the way you received your second medication review (i.e. face-to-face, over the telephone or video call)? *Please circle one of the following:*** | | | | | |
| Strongly like | Like | No opinion | Dislike | Strongly dislike | |
| 1. **Please briefly explain your reasons for stating this:** | | | | | |
| 1. **Did the doctor recommend any more changes to the medicines that you were taking at the time of the second medication review? *Please tick the appropriate box:*** | | | | | |
| Yes (If YES to Question 18, please complete Questions 19 & 20) | | | | |  |
| No (If NO to Question 18, please go to Question 21) | | | | |  |
| 1. **Did you agree with the doctor’s recommended change(s) to the medicines that you were taking at the time of the second review? *Please tick one of the following:*** | | | | | |
| Yes | | | | |  |
| No | | | | |  |
| 1. **Please briefly explain your reasons for agreeing/not agreeing with the change(s) that the doctor recommended:** | | | | | |
| **In the following questions, we would like you to think about both medication review appointments you received as part of the PolyPrime study.** | | | | | |
| 1. **Did you like or dislike attending the medication review appointments? *Please circle one of the following:*** | | | | | |
| Strongly like | Like | No opinion | Dislike | Strongly dislike | |
| 1. **How much do you agree with the following statement? Based on my experience, the PolyPrime intervention is likely to improve how many medicines are prescribed for older people. *Please circle one of the following:*** | | | | | |
| Strongly agree | Agree | No opinion | Disagree | Strongly disagree | |
| 1. **What has been the effect of having your medicines reviewed by your GP? *Please tick all that apply:*** | | | | | |
| The number of medicines I take has decreased | | | | |  |
| The number of medicines I take has increased | | | | |  |
| I have a better understanding about the medicines I take | | | | |  |
| The number of times I take my medicines each day has decreased | | | | |  |
| The number of times I take my medicines each day has increased | | | | |  |
| I am happier about my medicines | | | | |  |
| I feel reassured that my medicines have been reviewed | | | | |  |
| I am still concerned about my medicines | | | | |  |
| It has made no difference, please briefly explain: | | | | |  |
| If there have been other effects, please briefly explain: | | | | |  |

| **Part 3 – Your overall experience of the PolyPrime study** | | | | | | |
| --- | --- | --- | --- | --- | --- | --- |
| 1. **How would you sum up your experience of the PolyPrime study? *Please circle one of the following:*** | | | | | | |
| Very good | Good | Average | Poor | Very poor | | |
| 1. **Please briefly explain your reasons for stating this:** | | | | | | |
| 1. **How much effort was required for you to take part in the PolyPrime study? *Please circle one of the following:*** | | | | | | |
| No effort at all | A little effort | No opinion | A lot of effort | | Huge effort | |
| 1. **Please briefly explain your reasons for stating this:** | | | | | | |
| 1. **What would have improved your overall experience of being involved in the PolyPrime study? *Please tick all that apply:*** | | | | | | |
| Being sent an appointment letter for my medication review appointments | | | | | |  |
| Longer appointment(s) | | | | | |  |
| Shorter appointment(s) | | | | | |  |
| Nothing, I was happy with the overall experience | | | | | |  |
| Improvements could be made but have not been listed above. I have the following suggestions that might lead to improvements: | | | | | |  |
| 1. **Would you recommend being involved in the PolyPrime study to a friend or family member? *Please tick one of the following:*** | | | | | | |
| Yes | | | | | |  |
| No | | | | | |  |

Please use the return addressed envelope provided (or the address on Page 1) to send the questionnaire back to the research team.

If you would like to speak further to the research team about your experience, then please contact them using the details on Page 1.
